# Advances in body composition: a 100-year journey

**DOI:** 10.1038/s41366-024-01511-9

**Published:** 2024-04-20

**Authors:** Steven B. Heymsfield

**Affiliations:** Pennington Biomedical Research Center, Louisiana State University System, Baton Rouge, LA USA

**Keywords:** Medical research, Diagnosis

## Abstract

Knowledge of human body composition at the dawn of the twentieth century was based largely on cadaver studies and chemical analyses of isolated organs and tissues. Matters soon changed by the nineteen twenties when the Czech anthropologist Jindřich Matiegka introduced an influential new anthropometric method of fractionating body mass into subcutaneous adipose tissue and other major body components. Today, one century later, investigators can not only quantify every major body component in vivo at the atomic, molecular, cellular, tissue-organ, and whole-body organizational levels, but go far beyond to organ and tissue-specific composition and metabolite estimates. These advances are leading to an improved understanding of adiposity structure-function relations, discovery of new obesity phenotypes, and a mechanistic basis of some weight-related pathophysiological processes and adverse clinical outcomes. What factors over the past one hundred years combined to generate these profound new body composition measurement capabilities in living humans? This perspective tracks the origins of these scientific innovations with the aim of providing insights on current methodology gaps and future research needs.

One-century ago the Roaring Twenties were in full swing, Europe was recovering from the destructive effects of World War I, and the Czech anthropologist Jindřich Matiegka had just published his classic treatise on the anthropometric fractionation of body mass into subcutaneous adipose tissue plus skin, skeletal muscle, skeleton, and remainder [1]. Up until Matiegka’s time information on human body composition, including adiposity, was sparse; the prevailing knowledge was built largely around earlier anatomic and chemical analyses of cadavers and excised tissues [2]. Investigators such as Camerer and Söldner had reported the chemical composition of fetuses as fat, nitrogen, major minerals, and water in 1900 [3], although similar notable experimental studies were published at a slow rate of only one or two every several years. Matters changed remarkably over the past one-hundred years: publications reporting human body composition studies are appearing in scientific literature at a rate of more than 3000 per year, totaling over 60,000 since Matiegka’s time.^1^

Matiegka introduced his “somatotechnique” as a means of evaluating human physical efficiency following the “Great War” [1]. Deaths were declining from infectious diseases, although combat-related famines were soon to follow during World War II [4] and protein-calorie malnutrition prevailed during the mid-twentieth century in developing countries [5]. The physical effects, morbidity, and mortality related to semistarvation attracted a global cadre of eminent scientists whose careers were founded on the metabolic and body composition effects of undernutrition. Classic studies conducted in the aftermath of World War II such as the innovative Minnesota Starvation Experiment [6] still strongly influence investigators exploring topics related to body composition, metabolism, and obesity in humans [7, 8].

By the mid-1970s a different pattern of major killer diseases emerged: obesity appeared on the horizon [9] and heart disease replaced infectious diseases as the leading cause of death in the U.S. [10]. Adiposity phenotyping methods were still in an early stage of development; Spivak had introduced measurement of specific gravity in 1915 as a potential human vital sign [11] and by the 1960s the hydrodensitometry method for body fat estimation was still considered “*far from accurate*” [12]. Keys’ 1972 study of optimum adiposity power-type indices [13] leading to widespread use of body mass index relied largely on the sum of two skinfolds to quantify body fat in 7043 men of diverse backgrounds; 429 of the men also had body fat measured by hydrodensitometry. Major publications, such as the 1975 Reference Man [14], collated earlier data from the many studies reporting normative values acquired from cadavers and anthropometric measurements that were a hallmark of this era.

The emergence of obesity and related chronic diseases in the 1970s [9] prompted a new wave of body composition research aimed at developing methods of phenotyping people for adiposity and associated health risks. A second wave of body composition research emerged following Rosenberg’s 1989 report identifying sarcopenia as a major health concern [15]; sarcopenic-obesity soon appeared in other publications [16]. Developing methods for quantifying total body and regional adipose tissue and skeletal muscle mass became a major focus of research with specialized laboratories opening worldwide [2] and periodic international conferences devoted solely to advances in body composition methods and findings [17].

The broad research and clinical interest in human body composition is matched with a wide range of available measurement methods introduced over the past century. These methods are described in hundreds of publications, but most owe their origins to fundamental scientific discoveries during the nineteenth and twentieth centuries. The conceptual foundations for at least four families of methods are based on the Nobel-Prize winning discoveries of X-rays by Roentgen in 1895, radioactivity by Becquerel in 1896, the stable isotope deuterium by Urey in 1931, and nuclear magnetic resonance by Rabi in 1938 [18]. These fundamental discoveries led to downstream development of dozens of new methods for evaluating human body composition in general and specifically components related to adiposity [2]. The major families of new body composition methods and their historical foundations are visually depicted along with a timeline in Fig. 1 and an expanded summary in Table 1.

**Fig. 1:**
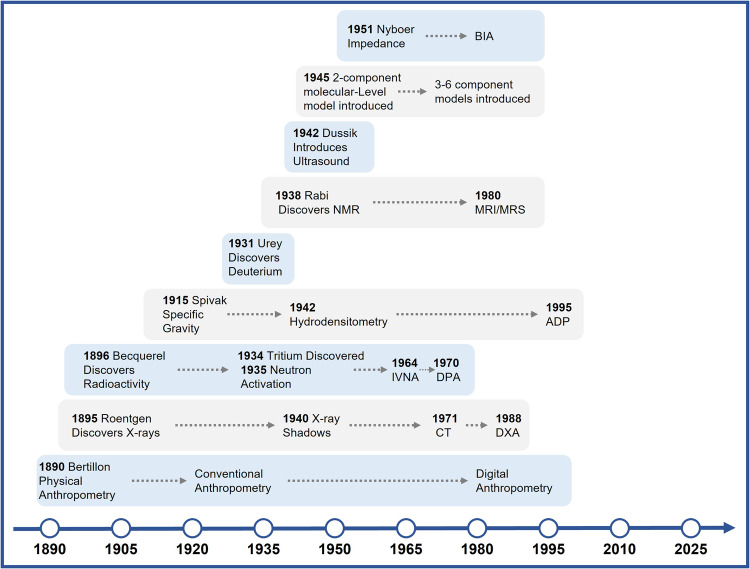
Chronological development of human body composition methods according to foundational discoveries and observations. Expanded comments are provided in Table 1. ADP air displacement plethysmography, BIA bioimpedance analysis, DPA dual-photon absorptiometry, DXA dual-energy X-ray absorptiometry, INVA in vivo neutron activation analysis, MRI magnetic resonance imaging, MRS magnetic resonance spectroscopy, NMR nuclear magnetic resonance.

**Table 1:** Historical pathways leading to development of human body composition methods.

Year	Innovation	Impact	Measured Components
1890	Bertillon introduces Physical Anthropometry [36]	Began as a forensic tool; 1921, Matiegka measured 4 body components [1]; 1989, Loughborough scanner begins era of digital anthropometry [37].	Whole-body and regional size, shape, and composition.
1895	Roentgen^a^ discovers X-rays [18]	1940, Stuart et al. use X-ray shadows for muscle and bone [38]; 1970s, Hounsfield^a^ and Cormack^a^ develop computed tomography (CT) [39, 40]; 1988, dual-energy X-ray absorptiometry (DXA) approved for clinical use [41].	CT: whole-body and regional tissue and organ volumes; selective organ/tissue composition; DXA: whole-body and regional fat, lean, and bone mineral mass.
1896	Becquerel^a^ discovers radioactivity [18]	1934, Rutherford^a^ & colleagues discover tritium [18, 42]; 1935, Hevesy^a^ & Levi introduce neutron activation concept [43]; 1964, Anderson et al. introduce in vivo neutron activation analysis (IVNA) [44]; 1970, Mazess et al. [45] report that γ-radiation at two photon energies can be used to separate tissues into fat, lean, and bone mineral components, an observation leading to dual-photon energy absorptiometry (DPA) and later DXA methods.	^3^H_2_O: total body water by isotope dilution; IVNA: all major body elements, from which molecular-level models can be created; DPA: whole-body and regional fat, lean, and bone mineral mass.
1915	Spivak measures specific gravity in humans [11]	1942, Behnke et al. relate specific gravity to adiposity [46]; 1995, McCrory et al. introduce air displacement plethysmography [47].	Whole-body fat and fat-free mass.
1931	Urey^a^ discovers deuterium [18]	1934, Hevesy^a^ & Hofer use deuterium (D_2_O) to measure total body water; introduce “isotope dilution” method [48].	D_2_O: total body water; isotope dilution method is used to measure multiple compartments.
1938	Rabi^a^ discovers Nuclear Magnetic Resonance (NMR) [49]	1946, Bloch^a^ and Purcell^a^ expand NMR techniques [50, 51]; 1970s, Lauterbur and Mansfield pioneered NMR for imaging; 1980, Damadian introduces first commercial magnetic resonance (MR) imaging scanner [52].	MR-Imaging and Spectroscopy: whole-body and regional tissue and organ volumes; selective organ/tissue composition and metabolites.
1942	Dussik introduces diagnostic ultrasound [53]	Medical applications evolved from earlier research in diverse fields [53].	Organ and tissue dimensions; selective organ/tissue composition.
1945	Molecular-level body composition Models introduced [54]	1945, Pace & Rathbun establish chemical stability of the fat-free body [54]; combinations of measured total body water, body density, and bone minerals over the following decades increased reported models from two to six.	2-component fat and fat-free mass model can be derived using multiple methods; more complex 3–6 component models often serve as the “reference” standard for body fat [55].
1951	Nyboer et al. introduce clinical impedance measurements [56]	1962, Thomasset [57]; and 1969, Hoffer et al. [58] correlate impedance with body water volume. Bioelectrical impedance scanners evolved from these early studies.	Depending on device, multiple whole-body and regional compartments.

^a^Scientists who won Nobel Prizes for the noted or related research in their respective fields.

These sweeping discoveries in the short span of five decades and the seminal observations of several other investigators led to the “golden era” of human body composition method development between the 1930s and 1980s. Every major body component at the atomic, molecular, cellular, tissue-organ, and whole-body levels can now be measured in vivo using methods fully developed over the past century. Components such as adipose tissue and skeletal muscle mass have been fully characterized and can now be assessed in the whole body and selected regions across the full lifespan with great accuracy and precision. An important advance over recent decades is that body composition measurements can now be extended far past assessments of whole component volume and mass to chemical and cellular compositions. Examples include brown adipose tissue within the total body adipose tissue component [19], intermuscular adipose tissue and intramyocellular lipids within skeletal muscles [20, 21], intrahepatic lipid in liver [22], white and gray matter in the brain [23], muscle microarchitecture [24], and vast numbers of tissue metabolites [21]. These kinds of capabilities extend traditional body composition analysis to estimation of an organ or tissues “quality” in vivo. Many of the features of organ and tissue quality have been linked with metabolic and physiological disturbances in people with obesity.

In-depth analysis of component volume or mass and composition bring us closer to a deeper understanding of structure-function relationships. An example is the condition associated with obesity referred to as heart failure with preserved ejection fraction (HFpEF) [25]. Not only can total heart volume and mass (a component at the organ-tissue body composition level) be measured non-invasively, but evaluation of detailed myocardial structure, metabolism, and function can be quantified with methods such as magnetic resonance imaging/spectroscopy and echocardiography. Moreover, cardiac structure and function can be mechanistically associated with total adiposity, visceral adipose tissue, epicardial adipose tissue, and myocardial steatosis along with metabolic factors such as the level of insulin resistance [25]. These observations were recently amplified when Kosiborod et al. [26] showed that reductions in adiposity following administration of a glucagon-like peptide-1 receptor agonist significantly improved multiple physical functions in patients with obesity and HFpEF. Building structure-function-outcome relationships such as these were inconceivable only several decades ago and portend the future of research into “functional body composition” as suggested by Muller [27]. Discovery of new phenotypes is possible [28–31], particularly with advancing mathematical capabilities for analyzing large and complex data sets increasingly available in stored cloud sites [32].

These notable advances that thrust body composition research into a much wider sphere of basic and clinical research also bring in scientists from other disciplines with fresh points of view and ideas. Their publications likely account for the surge in human body composition reports appearing over the last few years. However, we are not yet at the “end” of body composition methodology research [33]. Regretfully, we still don’t have highly accurate, practical, and low-cost body composition methods that are widely applicable in clinical settings, epidemiological surveys, and even at home. Normative values for many methods are lacking, notably across samples varying widely in age, sex, and race/ethnicity. Devices often acquire manufacturer-specific measurements and employ non-disclosed population-specific empirical prediction algorithms. Comparisons of adiposity estimates across patients and studies is thus difficult and often impractical. Not all body components and their composition are yet measurable; to note several, the mass of the large gastrointestinal tract and its subcomponents, pancreatic beta-cell mass, and mitochondrial mass with associated energy expenditure still cannot be accurately quantified in vivo by body composition researchers. As shown in Fig. 1, most of the available current body composition methods evolved from only a few basic or seminal discoveries and current advances stem largely from refinements rather than innovation. Thus, there is work ahead for those of us whose interests lie in developing and applying methods for quantifying body composition in vivo.

Going back one century, could Matiegka working with a flexible tape and rigid ruler [1] have envisioned the advances in body composition and adiposity evaluation that we can now marshal at research centers with ease? Could Keys and Brozek studying adiposity changes with semi-starvation one-half century ago [13, 34] have imagined their sophisticated hydrodensitometry system for evaluating body fat percentage would be all but extinct in 2024? How will artificial intelligence, already sweeping into analyses of body composition data [35], create a new quantitative paradigm for the field? Can we, working at the forefront of body composition research, visualize what the field will look like in 2124? While clearly the future cannot be accurately predicted by most of us, we can only hope that our scientific descendants will experience another golden age in body composition and obesity research.

## Footnotes

1 These data come from a PubMed search conducted January 2, 2024 using the search terms “human” and “body composition.”
